# Improved Hepatoprotective Effect of Liposome-Encapsulated Astaxanthin in Lipopolysaccharide-Induced Acute Hepatotoxicity

**DOI:** 10.3390/ijms17071128

**Published:** 2016-07-14

**Authors:** Chun-Hung Chiu, Chun-Chao Chang, Shiang-Ting Lin, Charng-Cherng Chyau, Robert Y. Peng

**Affiliations:** 1 Research Institute of Biotechnology, Hungkuang University, Taichung City 43302, Taiwan; chchiu@hk.edu.tw (C.-H.C.); n404user@gmail.com (S.-T.L.); 2 Division of Gastroenterology and Hepatology, Department of Internal Medicine, Taipei Medical University Hospital, Taipei 11031, Taiwan; chunchao@tmu.edu.tw; 3 Division of Gastroenterology and Hepatology, Department of Internal Medicine, School of Medicine, College of Medicine, Taipei Medical University, Taipei 11031, Taiwan; 4 Research Institute of Medical Sciences, Taipei Medical University, Taipei 11031, Taiwan

**Keywords:** Astaxanthin, liposome encapsulation, antioxidant enzymes, inflammation, hepatotoxicity

## Abstract

Lipopolysaccharide (LPS)-induced acute hepatotoxicity is significantly associated with oxidative stress. Astaxanthin (AST), a xanthophyll carotenoid, is well known for its potent antioxidant capacity. However, its drawbacks of poor aqueous solubility and low bioavailability have limited its utility. Liposome encapsulation is considered as an effective alternative use for the improvement of bioavailability of the hydrophobic compound. We hypothesized that AST encapsulated within liposomes (LA) apparently shows improved stability and transportability compared to that of free AST. To investigate whether LA administration can efficiently prevent the LPS-induced acute hepatotoxicity, male Sprague-Dawley rats (*n* = six per group) were orally administered liposome-encapsulated AST at 2, 5 or 10 mg/kg-day (LA-2, LA-5, and LA-10) for seven days and then were LPS-challenged (i.p., 5 mg/kg). The LA-10 administered group, but not the other groups, exhibited a significant amelioration of serum glutamic pyruvic transaminase (GPT), glutamic oxaloacetic transaminase (GOT), blood urea nitrogen (BUN), creatinine (CRE), hepatic malondialdehyde (MDA) and glutathione peroxidase (GSH-Px), IL-6, and hepatic nuclear NF-κB and inducible nitric oxide synthase (iNOS), suggesting that LA at a 10 mg/kg-day dosage renders hepatoprotective effects. Moreover, the protective effects were even superior to that of positive control *N*-acetylcysteine (NAC, 200 mg/kg-day). Histopathologically, NAC, free AST, LA-2 and LA-5 partially, but LA-10 completely, alleviated the acute inflammatory status. These results indicate that hydrophobic AST after being properly encapsulated by liposomes improves bioavailability and can also function as potential drug delivery system in treating hepatotoxicity.

## 1. Introduction

**Scheme 1:**
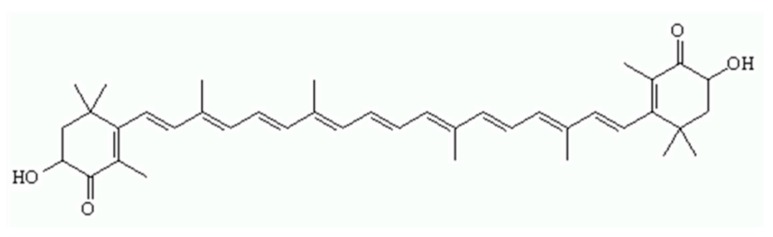
Chemical structure of astaxanthin.

Astaxanthin (3,3′-dihydroxy-β,β′-carotene-4,4′-dione) (AST, Scheme 1) is a xanthophyll carotenoid contained in *Haematococcus pluvialis*, *Chlorella zofingiensis*, *Chlorococcum*, and *Phaffia rhodozyma*. In fishery and poultry farms, AST is used to enhance the red coloration in salmon, trout, lobsters, shrimp, flamingoes and red ocean plants. Currently, much interest in finding the biological active compounds from microbial, plant and animal sources has been focused in the efficient targeting at molecular levels, which is involved in and associated with disease outcomes [1]. In vitro studies have indicated AST to be the compound that may uniquely exhibit all these characteristics. AST is well known for its potent antioxidant capacity [2], being much more powerful than vitamin C, E and β-carotene, and is often used in combined preparation with vitamin C, E and β-carotene as an antioxidant supplement [3]. AST showed potential biological activity in in vitro and in vivo models [1]. Regarding biomedical applications, AST is often used as a nutritional supplement, an antioxidant, an anticancer agent, and a preventive agent against diabetes, cardiovascular diseases, and neurodegenerative disorders to effectively stimulate immunity and suppress carcinogenesis due to its potent oxygen radical-scavenging activity and anti-carcinoma prognosis [1,4,5]. In addition, AST is a natural healthy remedy for sun-screening [3].

In oxidative stress–induced pathologies, it is thought that excessive production of reactive oxygen species (ROS) is associated with neurodegenerative diseases. AST has been found to have significant activity for reducing the ROS generation and exerting significant protective effects against the 6-hydroxydopamine (6-OHDA)-induced neuronal damage, which in part could be attributed to its potential antioxidant effects [6]. It has been demonstrated that AST triggered the CYP genes expressions involving CYP3A4 and CYP2B6 and subsequently induced the CYP P450–related enzymes [7], resulting in a very efficient in vivo antioxidative detoxification [7,8]. AST effectively attenuated chronic inflammatory diseases, gastrointestinal diseases, liver diseases, eye diseases, skin diseases, exercise-induced fatigue, and male infertility [5].

Lipopolysaccaride (LPS) challenge is useful for inducing the liver damage model. LPS may destroy the antioxidant system [9]. AST, an antioxidant marine carotenoid, restored physiological conditions in U937 cells stimulated with LPS (10 mg/mL) [9]. Pretreatment with AST (10 mM) for 1 h attenuated the LPS-induced toxicity and ROS production [9].

There are numerous studies of AST’s beneficial effects on biological functions being reported recently, including the potential activities in anti-inflammation, anticancer, antimelanogenesis, free radical–scavenging and singlet oxygen–quenching capabilities, as well as immune-enhancement activities [9]. In liver diseases, AST administered at 80 mg/kg per day revealed a significant protective effect on liver fibrosis [10] and reduced liver injury in ConA-induced autoimmune hepatitis [11]. However, the intractable treatment on liver fibrosis is involved in the origins of liver cells, in which stellate cell activation is the central event in hepatic fibrosis, complemented by other mediators, such as fibrocytes, fibroblasts and myofibroblasts, etc. The complex interactions among these cells cause irreversible scarring and persistent injury [12]. Moreover, a previous study has demonstrated that AST supplementation at 0.03% in the diet lowers the plasma concentrations of triacylglycerol (TAG), GOT and GPT, and increases the hepatic expression of endogenous antioxidant genes [13]. In our major interest, AST may exhibit a promising hepatoprotective effect [5,7,14]. However, the drawback in using AST is often due to its low aqueous solubility and extremely low oral bioavailability. Our previous study demonstrated that poor bioavailability of AST can be improved by liposomal encapsulation and it represents an effective agent for improving the stability and transportability of AST in hepatic cell lines [15]. Previously, liposome-encapsulation technology was used to study the diffusion, the trans-membrane transport rate and the consumption rate of the free AST (FA) and liposomal AST (LA). The three physical biochemical parameters were all found prevailingly improved for LA over that of FA. Thus, in order to improve the bioavailability and activity of AST, we prepared LA encapsulating different doses of AST. These two preparations (FA and LAs) were tested for their preventive capability against LPS-induced hepatotoxicity.

## 2. Results and Discussion

### 2.1. Change in Body and Organ Weights

There was no significant difference in body weight (BW) among the different groups. The initial body weight ranged from 254.5 ± 8.4 to 260.8 ± 10.0 g, and the final body weight increased to a range of 282.5 ± 7.5 to 310.5 ± 10.1 g. The liver weight (LW) ranged within 11.8 ± 1.1 to 14.1 ± 0.4 g, yielding a ratio of LW/BW within 4.2 ± 0.4 to 4.8 ± 0.5 g. The kidney weight (KW) ranged within 20.7 to 25.9 g giving a ratio of KW/BW within 0.08 to 0.1 (Table 1). Since there was no significant difference of the BW, KW, LW/BW ratio, and KW/BW ratio between any two groups (Table 1), the NAC, AST, and liposomal capsules were considered to be non-significant effects on the physical conditions of rats.

### 2.2. Field Emission Scanning Electron Microscope (FE-SEM) Image of the Liposomal Nanocapsules

**Figure 1:**
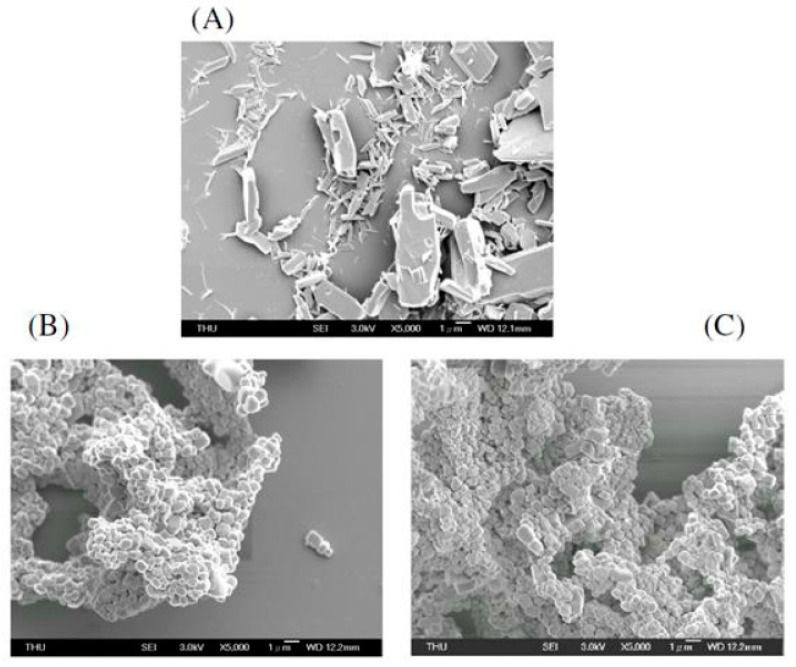
Representative field emission scanning electron microscope (FE-SEM) images of free Astaxanthin (AST), AST encapsulated within liposomes and free liposomes. Free AST (**A**); free liposome only (228 ± 32 nm) (**B**); and AST encapsulated within liposomes (LA) (240 ± 58 nm) (**C**) (magnification 5000×).

Free AST appeared randomly in crystalline forms of different size >26,000 nm (Figure 1A). Free liposomal nanoparticles exhibited a size distribution within 228 ± 32 nm (Figure 1B) while the AST-encapsulated liposomal particles showed a size distribution of 240 ± 58 nm (Figure 1C) (magnification 5000×), implicating the successful fabrication of the liposomal particles.

### 2.3. Level of Parameters Clinically Most Related to Liver Function

**Figure 2:**
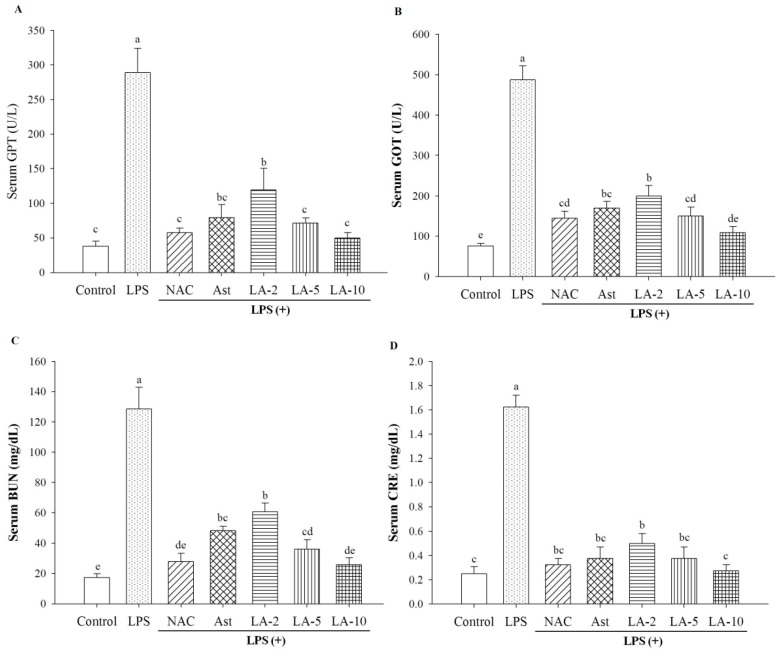
(**A**) Glutamate-pyruvate transaminase (GPT); (**B**) glutamate-oxaloacetate transaminase (GOT); (**C**) blood urea nitrogen (BUN) and (**D**) creatinine (CRE) levels in serum. Each bar represents the mean ± SEM of six rats per group. Statistical significance was determined using the one-way ANOVA with Tukey-Kramer post-hoc test. Different lower-case letters indicate statistically significant differences (*p* < 0.05). NAC, *N*-acetyl cysteine (200 mg/kg); LPS, lipopolysaccharide; LA-2, -5 and -10, liposomal AST at 2, 5 and 10 mg/kg BW, respectively.

The serum levels of glutamate-pyruvate transaminase (GPT), glutamate-oxaloacetate transaminase (GOT), blood urea nitrogen (BUN), and creatinine (CRE) were all stimulated by LPS insult to levels of 283 ± 40, 480 ± 30 U/L, 133 ± 33, and 1.62 ± 0.10 mg/dL (Figure 2), compared to their individual control values of 32 ± 8, 75 ± 10 U/L, 17 ± 1.5, and 0.22 mg/dL, respectively (*p* < 0.05) (Figure 2). *N*-acetylcysteine (NAC) at 200 mg/kg per day completely attenuated the level of GPT, but only moderately or almost completely restored the levels of serum BUN, serum CRE and GOT (*p* < 0.05) (Figure 2). Similar results were found for free AST (10 mg/kg per day) (Figure 2) (*p* < 0.05) (Figure 2). Amazingly, the liposome-encapsulated AST (LA-2 to LA-10) dose-responsively attenuated these serum parameters (Figure 2) and completely ameliorated them to normal levels by LA-10 (Figure 2) (*p* < 0.05), underlying the prevailing transport behavior of the liposome-encapsulated AST and the instantaneous pharmacokinetics of AST release from the nanoliposome particles.

Creatinine is a non-protein waste product of creatine phosphate degraded in muscle, and it is usually produced continuously and proportionally to muscle mass by the body. Creatinine levels in blood and urine may be used to estimate the creatinine clearance (CrCl), which reflects the glomerular filtration rate (GFR) of all functioning nephrons. The GFR is clinically important because it is a measurement of renal function. The BUN-to-creatinine ratio actually indicates other problems besides those intrinsic to the kidney.

### 2.4. Changes of Serum Nitrite, IL-6, and TNF-α

**Figure 3:**
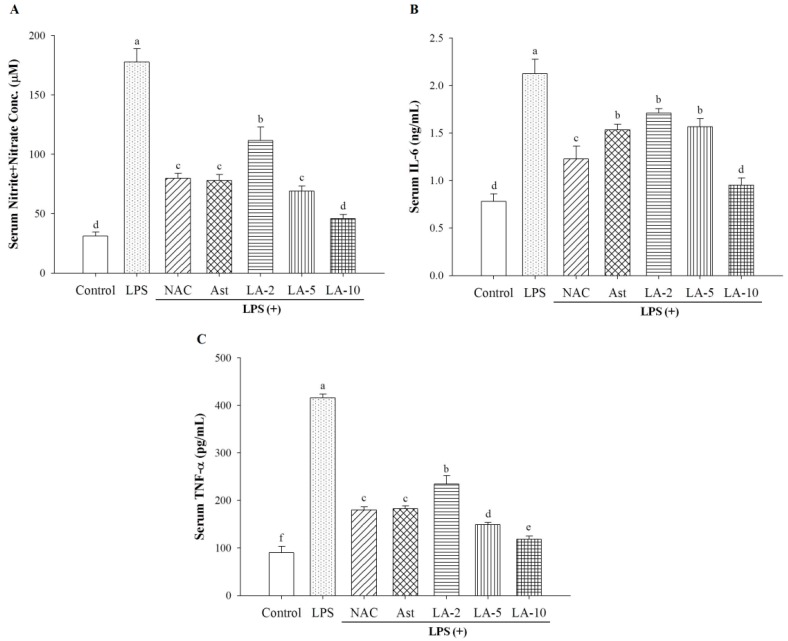
Nitric oxide (**A**), interleukin-6 (IL-6) (**B**) and tumor necrosis factor-α (TNF-α) (**C**) levels in the serum. Each bar represents the mean ± SEM (*n* = 6). Statistical significance was determined using the one-way ANOVA with Tukey-Kramer post-hoc test. Different lower-case letters indicate statistically significant differences (*p* < 0.05). NAC, *N*-acetyl cysteine (200 mg/kg); LPS, lipopolysaccharide; LA-2, -5 and -10, liposomal AST at 2, 5 and 10 mg/kg BW, respectively.

LPS highly induced the expression of serum nitrite, IL-6, and TNF-α to levels of 176 ± 10 µM, 2.1 ± 0.3 ng/mL, and 418 ± 18 pg/mL (*p* < 0.05), compared to the control values of 31 ± 3 µM, 0.6 ± 0.2 ng/mL, and 77 ± 20 pg/mL, respectively (*p* < 0.05) (Figure 3). NAC (200 mg/kg per day) and free AST (10 mg/kg per day) were only moderately effective for restoring these three parameters (Figure 3). LA-2 to LA-10, similar to the results in mentioned above, showed a dose-responsive effect; however, the alleviation was incomplete (Figure 3). The liposome-encapsulated AST dose-dependently attenuated levels of nitrite, IL-6, and TNF-α. Levels of nitrite and IL-6 were completely ameliorated by LA-10, while for TNF-α, all treatments failed to completely recover the elevations to control levels (Figure 3).

In inflammatory responses, the pro-inflammatory cytokine IL-6 was significantly induced after LPS stimulation (Figure 3). The production of IL-6 has been indicated from the stimulated vascular endothelial cells, macrophages, fibroblasts or monocytes [16]. In liver tissues, the different mediation effects of IL-6 are dependent on the sources of IL-6 [17]. IL-6, one of the predominant cytokines, activates STAT3 in hepatocytes and plays a significant role in liver protection and reconstruction, yet it can prompt the pro-inflammatory reaction in Kupffer cells via transient STAT3 activation. Reports suggested that IL-6 elicits STAT3 activation and which in turn facilitates the survival of cells [17]. Therefore, IL-6 could possess pro- and anti-inflammatory capabilities and the circulating IL-6 levels are directly and closely brought together with the progression of disease [18].

Tumor necrosis factor-α (TNF-α) has been suggested as a modulator of hepatotoxicity in the liver fibrosis model [19]. Blood levels of TNF-α in mice were raised by common bile duct ligation combined with cystic duct ligation. Amelioration of liver fibrosis but no improved effects on liver injury and hepatocyte regeneration were seen in TNF-α knock-out mice after common bile duct ligation and cystic duct ligation [19]. The increased expression levels of collagen α1(I) mRNA, transforming growth factor-β mRNA (TGF-β mRNA), and α-smooth muscle actin (αSMA) protein were independent of TNF-α after bile duct ligation and cystic duct ligation [19]. However, TNF-α produced by cholestasis can promote liver fibrosis via TIMP-1 production from hepatic stellate cells [19]. In our model, the serum TNF-α was seen highly stimulated by LPS to reach a level of 418 ± 18 pg/mL (Figure 3), implicating hepatic fibrosis can be provoked in similar ways.

However, inflammatory cascades driven by TNF-α play a major role in the progression of acute liver failure and its neurological complication [20]. Thus, modulating levels of TNF-α and TIMP-1 may constitute a new therapeutic strategy for treating hepatic fibrosis in cholestatic liver injury.

### 2.5. Histopathological Examination

**Figure 4:**
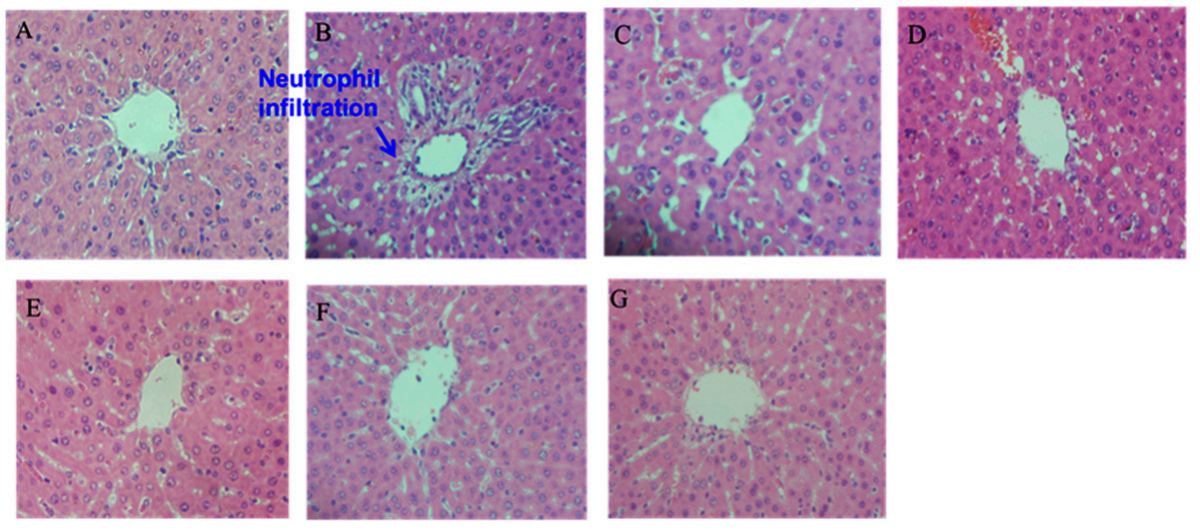
Histopathologic sections of liver tissues of control group (**A**); LPS group (**B**); LPS + NAC (200 mg/kg) (**C**); LPS + AST (10 mg/kg) (**D**); LPS + LA (2 mg/kg) (**E**); LPS + LA (5 mg/kg) (**F**); LPS + LA (10 mg/kg) (**G**). Magnification 400×.

LPS insult induced neutrophil infiltration in the hepatic cells (Figure 4B). Liposome-encapsulated AST 5 mg (LA-5) (Figure 4F) showed a better alleviative effect than the free AST (10 mg) (Figure 4C), being sufficient to ameliorate the neutrophilia caused by LPS insult. These results involved the reduction of TNF-α (Figure 3C), which has been recognized as one of the important cytokines in inducing the production of sepsis [18].

### 2.6. Oxidative Stress and Antioxidative Enzymes

**Figure 5:**
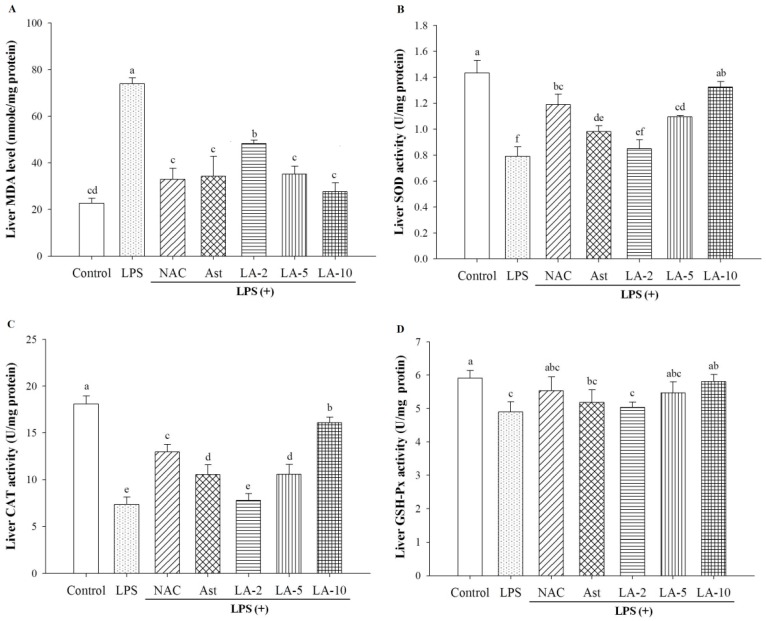
(**A**) Malondialdehyde (MDA), (**B**) superoxide dismutase (SOD), (**C**) catalase (CAT) and (**D**) glutathione peroxidase (GSH-Px) levels in liver. Each bar represents the mean ± SEM of three to four rats per group. Different letters indicate significant differences between groups. Statistical significance was determined using the one-way ANOVA with the Tukey-Kramer post-hoc test. Different lower-case letters indicate statistically significant differences (*p* < 0.05). NAC, *N*-acetyl cysteine (200 mg/kg); LPS, lipopolysaccharide; LA-2, -5 and -10, liposomal AST at 2, 5 and 10 mg/kg BW, respectively.

LPS induced a vast amount of hepatic malondialdehyde (hepatic MDA), reaching 73 ± 3 nmole/mg of protein compared to the control value of 22 ± 2 nmole/mg of protein. LPS severely suppressed the activity of hepatic superoxide dismutase (liver SOD) to 0.75 ± 0.25 U/mg of protein, hepatic catalase (liver CAT) to 7.2 ± 2 U/mg of protein, and hepatic glutathione peroxidase (liver GSH-Px) to 4.7 ± 0.5 U/mg of protein, respectively (Figure 5), compared to the control values of 1.41 ± 0.18 U/mg of protein (for SOD), 17.7 ± 2.2 U/mg of protein (for catalase), and 5.8 ± 0.5 U/mg of protein (for GSH-Px) (Figure 5), underlying the severely damaged antioxidative system of liver tissues by LPS. NAC and free AST were only moderately effective for rescuing the highly stimulated hepatic Malondialdehyde (MDA), superoxide dismutase (SOD), catalase (CAT) and glutathione peroxidase (GSH-Px) (Figure 5). Astonishingly, LA-10 almost completely alleviated the MDA, SOD and GSH-Px levels, and partially alleviated the CAT level. In contrast, the liposome-encapsulated AST (LA-2 and LA-5) only partially and dose-responsively rescued the levels of hepatic MDA and SOD (Figure 5). Results apparently implicated the more efficient and stable transport of encapsulated AST compared to free AST, and more importantly, they showed that through liposome encapsulation, a substantially higher intrahepatic uptake could be achieved.

Braithwaite et al. [21] reported similar findings that the incorporation of resveratrol into liposomal carrier systems has conferred improvements in biological activity and efficacy with an improved side effect profile, making possible oral and intravenous dosage formulations of the compound [21]. Literature elsewhere indicated that the nature of the environmental vehicles is the major contributor for both the antioxidant activity and free radical–scavenging power of AST [22]. In an octane/butyronitrile (9:1, *v*/*v*) system, AST was comparable to Trolox against peroxyl radicals, whereas in an emulsified dioleoylphosphatidyl choline liposomal suspension, AST exhibited superior capability as an antioxidant [3].

In the study of U937 cells stimulated with LPS (10 mg/mL), the LPS-induced toxicity and ROS production could be attenuated by the pre-treatment of AST (10 mM) for 1 h [9]. Reports indicated that a reduction in intracellular O_2_^●−^ production may be attributed to the beneficial effect of AST through the activation of antioxidant enzymes, which influence heme oxygenase-1 expression and activity by nuclear translocation of nuclear factor erythroid-2-related factor (Nrf2) [9]. Similar results in our experiment also revealed the upregulation of SOD by NAC, free AST and the liposome-encapsulated AST. The effect of 10 mg free AST was seen to be only comparable to LA-5, while LA-10 exhibited a far better effect in the alleviation of SOD, catalase, and GSH-Px (Figure 5). Previously, we showed that the consumption rate of free AST by Hep3B and HepG2 cells was negligible, while within the same duration of administration, that of the liposome-encapsulated AST reached 7.1 × 10^−7^ and 3.0 × 10^−7^ µg/h·cell, respectively, for these two cell lines [15]. The diffusion and transmembrane times were negligibly short for these two cell lines; in contrast, free AST took 0.5 h, while the consumption time was equal for the free AST and the liposome-encapsulated AST, which was 6.0 h. The overall transport time took 7.55 and 6.0 h, respectively, with the free AST and the encapsulated AST [15]. In addition, the liposome-encapsulated AST induced subG1 arrest in Hep3B and HepG2 cell lines [15]. Thus, the highly efficient delivery of the AST antioxidant in the circulating system using liposome encapsulation has proven the much improved overall therapeutic bioactivity in LPS-induced oxidative stress (Figure 5).

### 2.7. Expression of Nuclear iNOS and NF-κB

**Figure 6:**
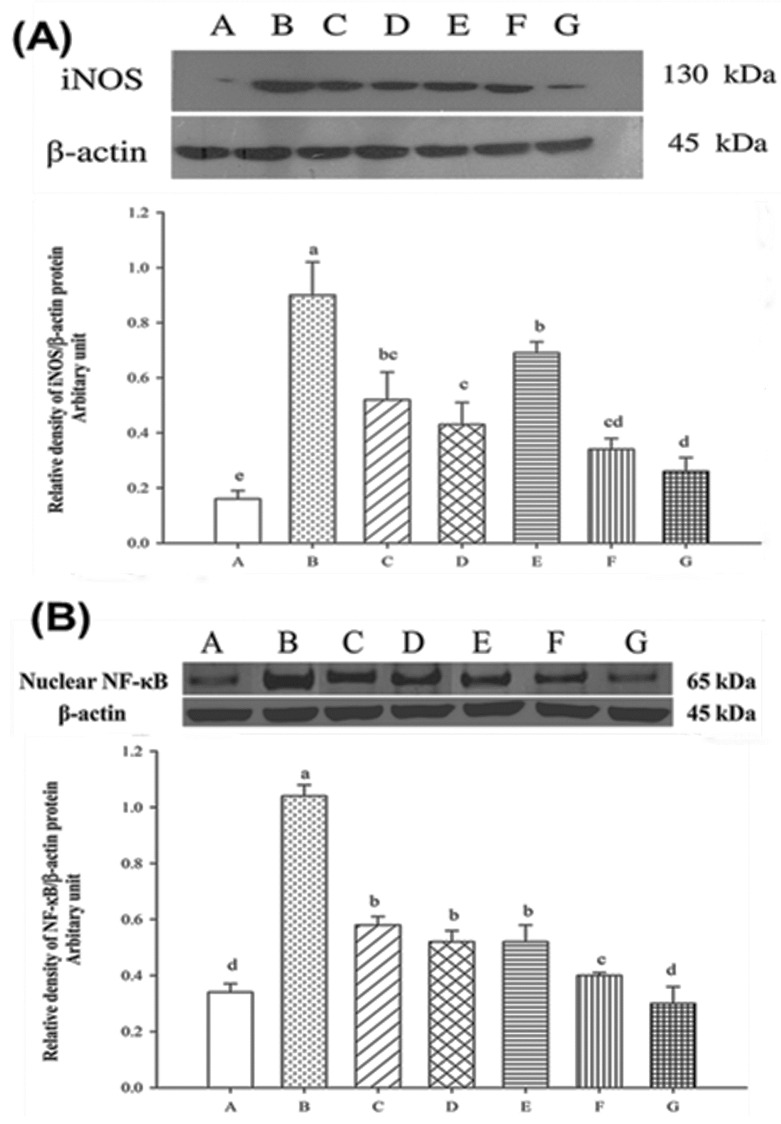
Expression of cytosolic iNOS (**A**) and nuclear NF-κB (**B**) on rat hepatic tissues. A: Control group, B: LPS group, C: LPS + NAC 200 mg/kg, D: LPS + AST 10 mg/kg, E: LPS + LA 2 mg/kg, F: LPS + LA 5 mg/kg, G: LPS + LA 10 mg/kg. Densitometric analyses of proteins presented as the relative ratio of each protein to β-actin. Values are means ± SEM of three independent experiments. Different lower-case letters indicate statistically significant differences (*p* < 0.05).

Levels of nuclear iNOS and NF-κB were highly expressed by LPS insult (Figure 6A,B). The liposome-encapsulated AST (LA-2 to LA-10) dose-dependently attenuated the elevated.

iNOS and NF-κB, and the effect of LA-10 was more effective than the free AST for both signals, again implicating the prevailing pharmacokinetic and pharmacodynamic characteristics of the liposome-encapsulated AST, i.e., a faster transport mechanism and a higher transit release rate of LA-10 in the hepatic tissues. Shen et al. reported that AST administered at 80 mg/kg per day revealed a significant protective effect on liver fibrosis by suppressing multiple profibrogenic factors and it significantly improved the pathological lesions of liver fibrosis [10]. The levels of GPT, GOT and hydroxyproline were significantly decreased by AST [10].

Why did AST act more efficiently than NAC in many respects? Both NAC and AST are powerful antioxidants. In addition to the antioxidative bioactivity, AST is able to inhibit the formation of extracellular matrix and the activation of hepatic stellate cells (HSCs) by reducing the expression of TGF-β1 and NF-κB and maintaining the between-matrix metalloproteinase 2 (MMP2) and metallopeptidase inhibitor 1 (TIMP1) [10]. Moreover, AST showed its ability to reduce the energy production in HSCs and downregulate the level of autophagy [10]. The in vivo and in vitro studies simultaneously verified the results [10].

The production of iNOS is dependent on the expression of IRF1 and NF-κB [23]. We showed LPS substantially upregulated the expression of NF-κB (Figure 6B), which in turn strongly stimulated iNOS (Figure 6A) to produce ·NO (Figure 3). Alternatively, iNOS produces large quantities of ·NO upon stimulation by proinflammatory cytokines such as IL-1, TNF-α, and interferon-γ [23]. Consistent with this, the level of serum TNF-α was highly upregulated by LPS treatment (Figure 3). Induction of high-output iNOS usually occurs in an oxidative environment (Figure 5), and thus high levels of ·NO have the opportunity to react with superoxide, leading to peroxynitrite (ONOO^−^) formation and cell toxicity.

In addition, iNOS may play a role in host immunity, enabling its participation in antimicrobial and antitumor activities as part of oxidative bursts of macrophages [24]. Nitric oxide (·NO) can modulate the response to respiration, biogenesis and hypoxia, depending on its concentration, through both mitochondria-dependent and mitochondria-independent pathways [25,26,27,28,29]. Higher concentrations of ·NO can cause irreversible inhibition of the respiratory chain, permeability transition, and/or cell death [25]. A superoxide/peroxynitrite-mediated oxidative stress and its toxic metabolite peroxynitrite can cause a decrease in the mitochondrial transmembrane potential, resulting in mitochondrial dysfunction [30,31], inhibiting the mitochondrial respiratory chain and reducing intracellular ATP content, leading to energy failure and ultimately cell death [30,31,32]. Exposure to ·NO donors triggered apoptosis with chromatin condensation, nuclei fragmentation, and DNA laddering [30], implicating the toxicological effect of LPS to induce severe hepatic damage.

However, in parallel to a high increase in the level of MDA, LPS downregulated the activity of hepatic SOD, catalase, and GSH-Px (Figure 5), underlying, at the same time, the substantially stimulated formation of peroxynitrite. In contrast, as the serum levels of GPT, GOT, BUN and creatinine were all highly upregulated by LPS (Figure 2), implying severe hepatotoxicity.

Matés et al. [33] reported that the antioxidant enzymes SOD, CAT and GSH-Px play very important roles in the protection against lipid peroxidation and ROS production [33]. The increased production of endogenous ROS may induce the increase of lipid hydroperoxides [33] (Figure 5A). Lipid hydroperoxide levels were negatively correlated with SOD and GSH-Px activities, as well as positively correlated with catalase activity [33]. Contrary to Matés et al. [33], the levels of SOD, CAT, and GSH-Px were all suppressed after treatment of LPS (Figure 5). However, these antioxidant enzyme levels in the livers were all ameliorated from the feeding of LA for seven days prior to the LPS challenges (Figure 5).

The excess production of ROS can cause a serious modification in the structure of biomolecules, such as deoxyribonucleic acid (DNA), proteins, lipids and carbohydrates. The occurrence of oxidative DNA damage may take part in ROS-induced carcinogenesis [34]. In the blood of patients with premalignant (hyperplastic) and malignant (adenocarcinoma) lesions, unbalanced antioxidant status was significantly presented, compared to those with benign uterine changes such as polyps and myoma [35]. Therefore, the homeostasis of the redox state is critical for maintaining normal physiological function.

## 3. Materials and Methods

### 3.1. Chemicals

Lipopolysaccharide (LPS), *N*-acetylcysteine, β-nicotinamide adenine dinucleotide 2′-phosphate reduced tetrasodium salt hydrate (NADPH), pyrogallol, 1,1,3,3-tetramethoxypropane (TMP), cholesterol and l-phosphatidylcoline (LPC, from soybean, ≥40%, thin layer chromatography, TLC) were purchased from Sigma Aldrich Co. (St. Louis, MO, USA). GSH reductase (1 U/mL), GSH, cholesterol and Griess reagent were products of Sigma Aldrich Co. Primary antibodies (iNOS, NF-κB, and β-actin, all 1:1000), phosphate buffered saline with Tween-20 buffer (PBST buffer containing 0.05% Tween-20) were purchased from Bio-Rad Co. (Hercules, CA, USA). Astaxanthin (>99%) was obtained as a donation from the Fuji Chemical Industry Co., Ltd. (Toyama Prefecture, Japan).

### 3.2. Methods

#### 3.2.1. Preparation of Liposome-Encapsulated Astaxanthin

The method for preparation of liposome-encapsulated astaxanthin was carried out as per our previous study [15]. Briefly, LPC (0.04 g/mL of DMSO) and cholesterol (0.01 g/mL of DMSO) were dissolved in 5 mL of mixed solvent of chloroform/methanol (2:1 *v*/*v*). To the mixture, an appropriately measured amount of AST was added. Finally, a sufficient amount of Smix was added to make the final concentration of AST to 1.0 mM. The solution was ultrasonicated and concentrated under reduced pressure to completely drive off the organic solvents until a membranous product appeared on the inner wall of the concentrator. The desiccation process was continued for additional 2 h in vacuum drier. The product membrane was redissolved in 15% of ethanol/double distilled water. The solution was sonicated for 30 min with a probe Sonicator XL2020 (25 W, 20 kHz; maximum rating power, 550 W; Heat System, Farmingdale, NY, USA). The dispersion was filtered through a 0.2-µm membrane (produced with the LiposoFast-Basic Extruder, Avestin Inc., Ottawa, ON, Canada). The filtrate was lyophilized to obtain LA.

#### 3.2.2. High-Performance Liquid Chromatographic (HPLC) Analysis for Astaxanthin Content in the Liposome-Encapsulated Particles

The LA (10 mg) was accurately weighed and transferred into a 100 mL beaker, to which 20 mL of PBS (pH 7.2, 30 °C) were added. The suspension was subjected to ultrasonication in the ultrasonic processor UP50H (50 W, 30 kHz (Hielscher Ultrasonics, Esquire Biotech, Teltow, Germany)). The ultrasonicated solution was filtered through a 0.22-µm filter. Twenty microliters of the filtrate was subjected to the HPLC analysis as described in previously report [15]. Briefly, for chromatographic separations, the Hitachi HPLC system (Tokyo, Japan) installed with a quaternary pump (Lachrom 7100), a diode-array detector (DAD, Lachrom 7455) and an injector (M-7725i Rheodyne) attached with 20 µL loop was used. The column packed with YMC™ Carotenoid S-5C30 (5 µm, Waters, Milford, MA, USA; 250 mm × 4.6 mm) was used to serve as the stationary phase. The mobile phase consisting of dichloromethane:water:acetonitrile:methanol in ratio of 17:2:12:69, *v*/*v* was operated in an isocratic elution mode at a flow rate of 1 mL/min. Astaxanthin detection in the DAD was carried out at 480 nm, and spectrum scans were covered from 320 to 700 nm. Quantification of AST was determined from a standard calibration curve established in parallel using known sample concentrations.

#### 3.2.3. FE-SEM Imaging of Liposome-Encapsulated Astaxanthin

Both the free liposomes and the LA was vacuum coated for 30 s with a gold atom layer. The morphology and size were examined at a resolution of 1.0 nm using the Field Emission Scanning Electron Microscopy (FE-SEM) (JEOL, model JSM-6500F, Tokyo, Japan).

#### 3.2.4. Animals and Treatments

The Institutional Animal Care and Ethics Committee of Hungkuang University (Taichung, Taiwan) approved all animal experiment protocols. Male Sprague-Dawley rats (six weeks old), weighing 265–287 g, were purchased from the BioLasCo Animal Center (Taipei, Taiwan). The rats, two rats in each stainless cage, were acclimated for one week on basic regular chow. The animal room was maintained at 21 ± 2 °C and 65% ± 5% relative humidity with a light/dark cycle of 12 h/12 h. The access to water and chow was ad libitum. At the beginning of the second week, the initial body weight was taken and these rats were divided into seven groups, six rats in each group based on body weight. Group 1 served as the negative control (saline control). Group 2, as the positive control, was treated with *N*-acetylcysteine (NAC) 200 mg/kg BW-day by gavage. Group 3 was treated with astaxanthin alone (10 mg/kg BW-day) per os. Groups 4 to 6 were treated respectively with liposome-encapsulated astaxanthin (LA) at 2, 5, and 10 mg/kg of BW per day by gavage. These different protecting treatments were continued for a period of 7 days. After the final BW was taken, rats were i.p. challenged with lipopolysaccharide (LPS) 5 mg/kg. After 12 h, rats were euthanized by CO_2_ inhalation and the bloods were collected from the hepatic portal vein and then centrifuged (3000 rpm, 10 min at 4 °C). The supernatant was used for determination of the levels of blood urea nitrogen (BUN), creatinine (CRE), glutamate-pyruvate transaminase (GPT), glutamate-oxaloacetate transaminase (GOT), nitrite (or ·NO), interleukine-6 (IL-6), and tumor necrosis factor-α (TNF-α). The livers and kidneys were excised and rinsed thrice with normal saline. The weight of organs was measured. Each liver was divided into four parts and immediately frozen if not in use. Liver tissues (part 1) were subjected to histopathological examination. Liver tissues of part 2 were homogenized with PBS and centrifuged at 3000× *g* for 20 min. The supernatant was used for determination of the levels of superoxide dismutase, catalase, and glutathione peroxidase. Livers of part 3 were used for protein extraction. The protein extract was used for Western blotting to exploit the change of iNOS level. Part 4 was frozen stored at −80 °C for further use.

#### 3.2.5. Assay for Serum GOT, GPT, CRE and BUN

Serum GOT, GPT, CRE and BUN were measured by colorimetric slides using the Fuji DRI-CHEM 3500s (Fujifilm Corp., Tokyo, Japan) to obtain various biochemical data.

#### 3.2.6. Assay for Serum Nitrite Level

Serum nitrite (including nitrate) concentration was measured by a modified method of the Griess assay, described by Miranda, Espey and Wink [36]. In brief, 100 µL of deproteinized serum samples were mixed with 100 µL of VCl_3_ and rapidly followed by the addition of Griess reagent (1% sulfanilamide dissolved in 5% phosphoric acid +0.1% naphthylethylenediamine dihydrochloride, 1:1, *v*/*v*). The absorbance was immediately measured at 540 nm after reacted for 15 min in dark at ABT. Authentic NaNO_2_ was used and similar protocol was applied to establish the calibration curve. From which the nitrite concentration was calculated and expressed as micromoles per liter.

#### 3.2.7. Assay for Serum IL-6 and TNF-α Levels

Levels of serum IL-6 and TNF-α were determined with rat IL-6 and TNF-α Quantikine solid phase sandwich ELISA kit, respectively. Briefly to a 96-well plate, 100 µL of capture antibody was added. The plate was tightly sealed and stored at 4 °C overnight. After several times of rinsing, the plates were blocked with Block Buffer at 25 °C for 1 h. Authentic IL-6, TNF-α or serum (100 µL) was added and left to react for 2 h at 25 °C. Dilute detection antibody was loaded to each well and incubated for an additional 2 h at ABT. Horseradish peroxidase (HRP) conjugated streptavidin (100 µL) was added and allowed to react for 30 min at 25 °C. A substrate solution (ABTS liquid substrate solution) (100 µL) was added and the mixture was allowed to react for 10 min in the dark. The absorbance was read at 405 nm using the ELISA reader (VersaMax, Molecular Devices, Sunnyvale, CA, USA). Linear calibration curves were determined similarly using authentic IL-6 and TNF-α.

#### 3.2.8. Hematoxylin-Eosin Staining (H & E Staining)

Part of liver tissue samples were cut into slices at a thickness of 0.5–1.0 cm and immersed in 10% neutral formalin solution for three days. Specimens were embedded in paraffin and frozen at 2–8 °C. The frozen tissues were sliced with a rotary microtome to yield slices having thickness of 4 µm, mounted onto a microscopic glass slide and treated with 50–60 °C water. After being tempered overnight in an oven held at 37 °C, the slices were stained with H & E, sealed with fat-soluble gel, and examined microscopically.

#### 3.2.9. Analysis of the Hepatic Malondialdehyde Level

The hepatic tissue thiobarbituric acid reactive substances (TBARS) level was determined as previous described [37] with slight modifications. Briefly, to 0.5 g of liver tissue, 5 mL of normal saline was added and homogenized. To determine the TBARS level, 2 mL of homogenate was added into the aliquots of 1.5 mL 0.01 N HCl, 0.5 mL 15% trichloroacetic acid (TCA), 0.375% thiobarbituric acid (TBA) and 0.25 N HCl, and was mixed well. The mixture was heated to 100 °C for 15 min. After cooling, the supernatant was separated by centrifugation at 3000× *g* for 10 min and was read at 535 nm. A calibration curve was established using TMP as the reference compound and analyzed similarly, from which the hepatic tissue TBARS level was calculated.

#### 3.2.10. Protein Extraction and Quantification

To determine the protein content in liver tissues, the Bradford method was followed [38]. To 0.5 g of hepatic tissue, a 10-fold volume of phosphate-EDTA buffer (0.1 mM, pH 7.0) was added, homogenized for 20 min, and centrifuged at 3000× *g* for 15 min. One mL of the supernatant was transferred to a micro-centrifuge tube and centrifuged at 4 °C and 12,000× *g* for 10 min. The protein content of the supernatant was determined (hereafter named homogenized hepatic extract, HHE).

#### 3.2.11. Assay for Hepatic Antioxidant Enzymes

The method of previous report [39] was followed to determine the hepatic superoxide dismutase (SOD) activity. Briefly, to 40 µL of HHE-Tris-phosphate EDTA buffer (50 mM, pH 7.0, 56 µL), a Triton X-100 solution (2%, pH 8.2, 96 µL) was added, mixed well, and centrifuged at 4 °C and 12,000× *g* for 5 min. Ten µL of the obtained supernatant was added into 3 mL of Tris-HCl (pH 8.2, 50 mM) and 10 µL of the methanolic pyrogallol (50 mM). The aliquot was immediately mixed, and read at 325 nm. Readings were successively made every 15 s for a total period of 3 min. A blank was similarly conducted using purified water. The activity was expressed in U/mg of protein. Catalase (CAT) activity was measured at 25 °C by reading the H_2_O_2_ (30 mM) decomposition successively every 15 s for a total period of 3 min. The hepatic catalase activity is expressed as U/mg of protein [40]. Hepatic glutathione peroxidase (GSH-Px) activity was determined by the method of Lawrence & Burk [41] with slight modifications. In brief, 100 µL of the obtained supernatant as described above was added into 800 µL of potassium phosphate buffer solution (containing 1 mM EDTA, 1 mM NaN_3_, 0.2 mM NADPH, 1 U/mL GSH reductase and 1 mM GSH). To this reaction mixture, 100 µL of 2.5 mM H_2_O_2_ was added and mixed well. The optical density (OD) was read at 340 nm. Readings were successively taken every 5 s for a period 3 min. The GSH-Px activity is expressed as U/mg protein.

#### 3.2.12. Extraction of the Cytosolic Proteins from Hepatic Tissues

Lysis buffer, in composed of 50 mM Tris-HCl pH 7.4, 1% Triton X-100, 150 mM NaCl, 1 mM EDTA, 0.1% SDS, 1 mM phenylmethanesulfonylfluoride (PMSF) and 1% sodium deoxycholate, was mixed with protease inhibitor (100:1, *v*/*v*, lysis buffer-protease inhibitor, LB-PI). To 200 mg of hepatic tissues were lysed in 1 mL of ice-cold LB-PI for 30 min. The lysate was centrifuged at 14,000× *g* for 20 min at 4 °C. The supernatant was separated and stored at −80 °C for further use. The pellet residue was used for extraction of the nuclear proteins.

#### 3.2.13. Extraction of Nuclear Proteins Extraction

The pellets were mixed with ice-cold 400 μL of 10 mM Tris-HCl (pH 7.5, containing 10 mM NaCl) for 10 min. The aliquot was centrifuged at 4 °C and 1300× *g* for 10 min. The precipitate was separated and extracted with 150 μL of ice-cold HEPES buffer (50 mM, pH 7.5, containing 0.5 mM EDTA, 0.1 mM EGTA, 420 mM NaCl and 10% glycerol) under the sonication for 1 min, and centrifuged at 4 °C and 10,000× *g* for 10 min. The supernatant was separated and stored at −80 °C for use.

#### 3.2.14. SDS-PAGE and Protein Transfer

The prepared proteins underwent SDS-PAGE at 7.5% of gel. A potential 100 V was applied, and the electrophoresis was started for 90 min. After the electrophoresis, the SDS-PAGE gel was immersed into the transfer buffer (pH 9.0–9.4, 50 mM Tris, 40 mM glycine, 0.375% SDS and 20% methanol). Alternatively, the polyvinylidene fluoride (PVDF) membrane was immersed in methanol for 5 min and then together with filter paper immersed in transfer buffer. Protein transfer was conducted in the wet transfer chamber (Bio-Rad) at 10 V for 60 min to carry out the transfer of protein onto the PVDF membrane. The PVDF membrane was peeled off and dipped into 5% skimmed milk in TBST buffer (1× TBS, 0.1% Tween-20) at 25 °C for 90 min.

#### 3.2.15. Antibody-Antigen Binding

The PVDF membrane created by the protein transfer of cytosolic and nuclear proteins was immersed in primary antibodies (iNOS, NFκB, and β-actin, all 1:1000) for 2 h at 25 °C and then rinsed three times with PBST buffer (containing 0.05% Tween-20), each time for 10 min. The rinsed PVDF membrane was immersed in the enhanced chemiluminescence (ECL) system solution and the relative signal intensity was by densitometric scanning of the blots and analyzed using ImageMaster^TM^ 2D platinum software (GE Healthcare, San Francisco, CA, USA).

### 3.3. Statistical Analysis

All values are expressed as the mean ± standard error of mean (SEM). Statistical significance was determined using one-way analysis of variance (ANOVA) with Tukey-Kramer post-hoc comparison test. A value of *p* < 0.05 was considered statistically significant.

## 4. Conclusions

The poor bioavailability of astaxanthin in reality has been improved by the liposome-encapsulation technology. Our work implicates a general rule stating that the bioavailability of any lipophilic active principles of precious therapeutic value, such as astaxanthin, in fact can be enhanced by such liposomal encapsulation. This experiment uncovers the fact that the liposome-encapsulated AST (LA) can exhibit highly stable circulation in the blood and efficient uptake by the liver, enhancing intrahepatic cellular antioxidant enzyme activities, leading to a more effective antioxidative effect by attenuating the LPS-suppressed SOD, catalase and GSH-PX, as well as more efficient anti-inflammatory bioactivity against the proinflammatory markers NO, IL-6 and TNF-α. In addition, LA exhibits a more promising downregulating effect on hepatic nuclear NF-κB and iNOS. As a consequence, LA (10 mg/kg per day) would show the most promising protective effect in attenuating hepatotoxicity when used as a clinical therapy to treat hepatic inflammatory damage.

## Figures and Tables

**Table 1:** The body and tissue weight measurements in each group ^1^.

Parameter	Control	LPS	NAC + LPS	Ast + LPS	LA-2 + LPS	LA-5 + LPS	LA-10 + LPS
Body weight (g) Initial Final	254.50 ± 8.35 ^a^ 298.12 ± 6.76 ^a^	259.25 ± 4.57 ^a^ 302.00 ± 2.58 ^a^	258.50 ± 8.58 ^a^ 282.50 ± 7.54 ^b^	260.75 ± 10.01 ^a^ 295.75 ± 4.99 ^a^	259.75 ± 2.21 ^a^ 302.25 ± 9.21 ^a^	259.50 ± 6.24 ^a^ 303.50 ± 7.14 ^a^	257.75 ± 7.32 ^a^ 310.50 ± 10.14 ^a^
Liver weight (g)	12.75 ± 0.64 ^a^	12.62 ± 2.30 ^a^	11.75 ± 1.07 ^a^	14.02 ± 1.34 ^a^	12.55 ± 0.60 ^a^	13.05 ± 1.04 ^a^	14.07 ± 0.41 ^a^
Liver weight/Body weight (%)	4.41 ± 0.21 ^a^	4.21 ± 0.36 ^a^	4.15 ± 0.31 ^a^	4.84 ± 0.46 ^a^	4.15 ± 0.27 ^a^	4.29 ± 0.26 ^a^	4.53 ± 0.12 ^a^
Kidney weight/Body weight (%)	0.89 ± 0.11 ^a^	0.95 ± 0.12 ^a^	0.81 ± 0.13 ^a^	0.87 ± 0.28 ^a^	0.91 ± 0.07 ^a^	0.91 ± 0.03 ^a^	0.90 ± 0.05 ^a^

^1^ Data were presented as the mean ± SEM (*n* = 6) and analyzed by one-way ANOVA with Tukey-Kramer post-hoc test. Means with different superscript lettered labels within a row are significantly different from each other (*p* < 0.05). LPS, lipopolysaccharide; Ast, astaxanthin; LA-2, -5 and -10, liposomal Ast at 2, 5 and 10 mg/kg BW, respectively.

## Abbreviations

AST: Astaxanthin; LA: Liposome-encapsulated AST; LPS: Lipopolysaccharide; GPT: glutamic pyruvic transaminase; GOT: Glutamic oxaloacetic transaminase; BUN: Blood urea nitrogen; CRE: Creatinine; MDA: Malondialdehyde; GSH-Px; Glutathione peroxidase; IL-6: Interleukin 6; NF-κB: Nuclear factor-κB; iNOS: Inducible nitric oxide synthase; NAC: *N*-Acetylcysteine.
